# Brain Knowledge and the Prevalence of Neuromyths among Prospective Teachers in Greece

**DOI:** 10.3389/fpsyg.2017.00804

**Published:** 2017-05-29

**Authors:** Marietta Papadatou-Pastou, Eleni Haliou, Filippos Vlachos

**Affiliations:** ^1^Faculty of Primary Education, Research Center for Psychophysiology and Education, School of Education, National and Kapodistrian University of AthensAthens, Greece; ^2^Cognition and Health Research Group, Department of Experimental Psychology, University of OxfordOxford, United Kingdom; ^3^Department of Special Education, University of ThessalyVolos, Greece

**Keywords:** neyromyths, educational neuroscience, teachers, special education, neuroscience

## Abstract

Although very often teachers show a great interest in introducing findings from the field of neuroscience in their classrooms, there is growing concern about the lack of academic instruction on neuroscience on teachers' curricula because this has led to a proliferation of neuromyths. We surveyed 479 undergraduate (mean age = 19.60 years, *SD* = 2.29) and 94 postgraduate students (mean age = 28.52 years, *SD* = 7.16) enrolled in Departments of Education at the University of Thessaly and the National and Kapodistrian University of Athens. We used a 70-item questionnaire aiming to explore general knowledge on the brain, neuromyths, the participants' attitude toward neuroeducation as well as their reading habits. Prospective teachers were found to believe that neuroscience knowledge is useful for teachers (90.3% agreement), to be somewhat knowledgeable when it comes to the brain (47.33% of the assertions were answered correctly), but to be less well informed when it comes to neuroscientific issues related to special education (36.86% correct responses). Findings further indicate that general knowledge about the brain was found to be the best safeguard against believing in neuromyths. Based on our results we suggest that prospective teachers can benefit from academic instruction on neuroscience. We propose that such instruction takes place in undergraduate courses of Departments of Education and that emphasis is given in debunking neuromyths, enhancing critical reading skills, and dealing with topics relevant to special education.

## Introduction

Neuroscience literacy amongst the general public (e.g., Herculano-Houzel, 2002) and specifically amongst teachers has been receiving increasing attention (e.g., Dekker et al., 2012; Deligiannidi and Howard-Jones, 2015). Teachers are showing great interest in the advances of neuroscience and in translating neuroscientific findings into their classrooms (e.g., Pickering and Howard-Jones, 2007; Zambo and Zambo, 2009, 2011; Bartoszeck and Bartoszeck, 2012; Serpati and Loughan, 2012; Rato et al., 2013; Karakus et al., 2015). At the same time, there is growing concern about the limited knowledge of brain facts and the rapid proliferation of neuromyths among teachers (e.g., Goswami, 2006; Pasquinelli, 2012).

The term “brain—compatible teaching” was first put forward in 1978 (Hart, 1978), who suggested that neuroscientific developments could provide a radical new way of looking at learning with enormous potential for helping teachers bring about major gains in their students' achievement. However, in 1997 Bruer argued that education and neuroscience are “a bridge too far.” He claimed that the distance between the two disciplines was too far to make meaningful extrapolation from neuroscience to educational application, and that this distance could only be covered with the introduction of a third discipline, psychology (Bruer, 1997). Since then, a number of publications have argued that education can be informed by neuroscience, as many believe that the findings from brain research can be transformed into practical strategies teachers could use to improve their teaching (e.g., Geake and Cooper, 2003; Goswami, 2004; Blakemore and Frith, 2005; Posner and Rothbart, 2005; Ansari and Coch, 2006; Immodino-Yang and Damasio, 2007; Pickering and Howard-Jones, 2007; Varma et al., 2008; Howard-Jones, 2014; Ansari, 2015; but also see Willingham, 2009; Horvath and Donoghue, 2016, for more skeptical accounts). It has even been claimed that an interface can be constructed between educational psychology and cognitive neuroscience, with the benefits of this interface being comparable to those accrued when a paradigm shift from a behaviorist orientation to a cognitive perspective in the 1960s and 1970s took place (Byrnes and Fox, 1998).

However, neuroscience is a complex field. The difficulty in understanding neuroscience findings creates fertile ground for a number of misinterpretations and of course neuromyths creation (Goswami, 2006). The fact that findings do not lend themselves to direct implementation in the classroom (Jolles et al., 2006; Devonshire and Dommett, 2010; Ansari et al., 2011) coupled with flaws in the media coverage of neuroscientific discoveries further feeds the proliferation of neuromyths (Pasquinelli, 2012). Even though there is a grain of truth in some of the myths, when revisiting the original research one can appreciate that it usually describes animal studies or that it has been oversimplified [Organisation for Economic Co-operation Development, 2002]. The term “neuromyths” was first used by neurosurgeon Alan Crockard in the 1980s to refer to unscientific ideas about the brain in medical culture (Crockard, 1996). It was re-defined in 2002 as “a misconception generated by a misunderstanding, a misreading, or a misquoting of facts scientifically established (by brain research) to make a case for use of brain research in education and other contexts” (Organisation for Economic Co-operation Development, 2002). The proliferation of neuromyths amongst teachers is worrisome, as the adoption of such myths wastes money, time, and energy resources that could be rather spent on evidence-based practices.

### How can neuroscience inform education

In 2008 the Society for Neuroscience published a list of “Neuroscience Core Concepts” that are relevant to educational practice for K-12 teaching (Society for Neuroscience, 2008). These include ideas such as “The brain is the most complex organ,” “Life experiences change the nervous system,” “Intelligence arises as the brain reasons, plans, and solves problems,” and “The brain makes it possible to communicate knowledge through language.” In 2011, the Royal Society stated that “educational practice can be transformed by science, just as medical practice was transformed by science about a century ago” (p. 3; Royal Society, 2011). According to the report, this could be done in a number of ways, for example, by showing that biological factors play an important role when it comes to individual differences in learning ability, by uncovering why certain types of learning are more rewarding than others, by showing that resilience can be build up through education, and by investigating the means of boosting brain power (Royal Society, 2011).

Findings from the field of neuroscience can further help teachers in a number of other ways. Dubinsky et al. (2013) suggested that the concept of plasticity can directly transform teacher preparation and professional development and motivate students to learn, even without the medium of psychology. In addition, Dubinsky et al. (2013) proposed that neuroscience experiments can reinforce prior results of psychological studies -that currently inform education- by providing biological explanations. For example, research findings, such as those that report increases in left occipitotemporal activation during a phonological reading intervention (Shaywitz et al., 2004), might provide the brain basis of phonics use in reading instruction. Recent neurocognitive data from the field of mathematics education provide ground for educational interventions, such as board and computer games, which have been found to benefit the numerical development in both typically and atypically developing students (De Smedt et al., 2013). Furthermore, knowledge about the development of the nervous system can support teachers in understanding their students' behavior, for example the risk-taking behaviors often observed in adolescents. Neuroscience can also explain how life-style decisions, such as exercise (Voss et al., 2011; for a review see Gearin and Fien, 2016), nutrition (Ivanovic et al., 2004), and sleep (Wang et al., 2011) can support learning.

It has further been claimed (Berninger and Richards, 2003) that teachers not only need to know the basics facts of neuroscience, but that they have the right or even the responsibility to know. Teachers work closely with other professionals, such as physicians, therapists, and audiologists, who know about the brain through their training. Therefore, in order for the different professionals to communicate, they need to be able to speak the same language. While other professionals might need to learn more about education, teachers also need to learn about neuroscience; only then will different professionals be in a position to efficiently collaborate in order to develop appropriate support strategies for each student's individual needs. Moreover, it will allow for the critical evaluation of the suggestions made by other professionals. At the same time, neuroscience literacy may help teachers avoid the pitfalls of commercial products, such as the Brain Gym© (Dennison and Dennison, 1994) and the VAK approach (e.g., Smith, 1996). Such products are claimed to have been developed based on neuroscientific findings, but in reality lack scientific back up (e.g., Coffield et al., 2004; Krätzig and Arbuthnott, 2006; Waterhouse, 2006; Stephenson, 2009; Pashler et al., 2010; Spaulding et al., 2010; Sylvan and Christodoulou, 2010; Witkowski, 2010; Lindell and Kidd, 2011). They are also costly not only financially, but also in terms of resources needed to apply them and time taken from other classroom activities.

### Neuromyths

Three examples of neuromyths are that “We only use 10% of our brains,” “There are right-brain and left-brain learners,” and that “Individuals learn better when they receive information in their preferred learning style (e.g., auditory, visual, kinesthetic)” (Organisation for Economic Co-operation Development, 2002; Geake, 2008; Purdy, 2008; Howard-Jones, 2010; Lindell and Kidd, 2011). Often, if not always, neuromyths are based on scientifically substantiated findings, which have been altered and have acquired a completely different meaning (Vlachos, 2010). For example, the myth of using only 10% of our brain could have stemmed from the fact that 10% of the brain consists of neurons, while the remaining 90% are glial cells. Of course, this does not mean that glial cells are not contributing to our brain function: they support and nourish neurons and recent evidence has even shown that they might even contribute information processing (Fields et al., 2014). The origin of the 10% neuromyth is hard to track down, but this myth is usually attributed to William James' statement “We are making use of only a small part of our possible mental and physical resource” (James, 1907), or to a misquote of Albert Einstein. When the popular media kept on repeated this statement the myth proliferated. The myth of right-brain and left-brain learners describes the popular belief that the left hemisphere is logical, whereas the right one is creative, with these qualities then attributed to people and to the way they learn (Organisation for Economic Co-operation Development, 2002). This myth is founded on the reality of hemispheric specialization, forgoing that such specialization is far from being absolute and that the brain is a highly integrated system (Organisation for Economic Co-operation Development, 2002; Papadatou-Pastou, 2011), but also that most of the work has been performed on patients whose corpus callosum, the structure that connects the two hemispheres, has been surgically severed (Gazzaniga, 1998). The learning styles myth claims that instruction ought to be tailored to the student's learning preference or more accurately to the student's learning style in order for the information to be more efficiently learned. This notion has been probably popularized as it implies that everyone can learn well, even equally well, if only the information to be learned matched their learning style; a notion that has become a virtual truism in education (Lilienfeld et al., 2011). However, there is no empirical support for style-based instruction (Rohrer and Pashler, 2012).

There is growing literature on the adoption of neuromyths amongst prospective or in-service teachers. Neuromyths seem to be adopted by teachers in the UK and the Netherlands, with teachers believing in 49% of the neuromyths in a cross-national survey of 242 primary and secondary school teachers (Dekker et al., 2012). Neuromyths were also adopted by 158 trainee teachers surveyed in the UK (Howard-Jones et al., 2009). Rato et al. (2013) surveyed 583 Portuguese teachers who taught in preschool to high school levels and found that they failed to distinguish myths from facts, irrespective of the area taught and level of teaching. In 2015, a number of surveys were published giving similar findings from other parts of the world. Gleichgerrcht et al. (2015) in a survey of 3,451 Latin American teachers reported that they also hold major misconceptions about neuroscience. Tardif et al. (2015) surveyed 44 in-service high-school teachers, 57 college teachers, 160 first-year primary student teachers, and 22 teacher's trainers in the French-speaking part of Switzerland and found that both teachers and student teachers believe in the reality of hemispheric and modality dominance. Karakus et al. (2015) surveyed 278 primary and secondary school teachers in Turkey and reported that Turkish teachers held many of the brain misconceptions that have been observed elsewhere, for example 97.1% of teachers believed that individuals learn better when they receive information in their preferred learning style. In East China, Pei et al. (2015) surveyed 238 teachers and identified many neuromyths popular elsewhere in the world, such as the learning styles myth, the myth of left-brained and right-brained learners and the myth of using only 10% of the brain. In Greece, Deligiannidi and Howard-Jones (2015) surveyed 217 primary and secondary school teachers and similarly reported the adoption of neuromyths among them, including the belief that differences in hemispheric dominance can help explain individual differences amongst learners (71% of the teachers agreed), and that teaching to learning styles is effective (97%). In Spain, 284 teachers from 15 independent Spanish regions were surveyed (Ferrero et al., 2016) and they were found to also endorse neuromyths, with 91.1% of the teachers believing in the learning styles myth.

### Factors affecting the adoption of neuromyths

Previous work has tried to answer the question of which factors predict the adoption of neuromyths. Howard-Jones et al. (2009) reported that general knowledge about the brain acted as a protective factor in their sample of prospective teachers. Moreover, Herculano-Houzel (2002) found that the reading of popular science magazines and newspapers were main contributors in improving neuroscience literacy. However, these findings are in contrast with the Dekker et al. (2012) findings that showed that general knowledge predicted increased belief in neuromyths. Of note, only teachers who reported an interest in neuroscience were included in the Dekker et al. (2012) sample. More recently, Gleichgerrcht et al. (2015) similarly found that teachers in Latin America who were found to know more about the brain, were more likely to believe in neuromyths.

The internet also abounds with web sites related to the brain that are of questionable validity -or plain wrong-, for example, websites that claim they can raise intelligence scores, cure attention deficits, and make babies more motivated to learn (Zambo and Zambo, 2009). This is particularly worrisome, as 64.2% of the 215 prospective teachers surveyed by Zambo and Zambo (2009) claim to be currently using the internet as a source of information and this number is expected to go up in the future. Moreover, media coverage of neuroscientific studies usually provides a deceptively simplistic or overly exaggerated interpretation of results, often accompanied by a misguided confidence in biological data (Beck, 2010). According to Pasquinelli (2012) there are three major flaws in the media coverage of neuroscientific discoveries: omission of relevant information on how results are obtained and brain images produced, sensationalism, and irrelevant information. In order to critically evaluate the reported findings, be it on the web or in the press, teachers need to have developed an understanding of not only the workings of the nervous system, but also of how neuroscientific research is carried out. For example, it is important to understand that findings are often based on averages and that they might not be applicable to every student individually or that studies that have used adults as participants (let alone non-human animals) may not be readily generalizable to younger populations, such as their students (Ansari, 2015). Interestingly, research has shown that people tend to believe research findings when they are accompanied by brain images and/or neuroscientific explanations (McCabe and Castel, 2008; Weisberg et al., 2008; but also see Michael et al., 2013, who failed to replicate these effects), even when those explanations are pseudo-scientific or plain irrelevant to the topic at hand. The attitude of believing that an image of the brain is sufficient to prove the existence of a mental state has been termed “neurorealism” (Racine et al., 2006).

### Special education

While neuroscience literacy amongst teachers has been documented in a number of countries, neuroscientific knowledge that taps specifically on special education has been largely overlooked. This knowledge could help special education teachers better understand not only the causes of neurodevelopmental disorders, but also the process of learning in non-typically developing children and direct them to best practices. Moreover, neuroscience findings can contribute to the early identification of learning difficulties (Goswami, 2009) and the development of evidence-based interventions.

For example, there is growing literature on phonological interventions for students with dyslexia, which not only enable students to improve phonological decoding skills, but result in atypical brain activation profiles to return to typical patterns, comparable to those of typically developing children (Simos et al., 2002; Shaywitz et al., 2004; Spironelli et al., 2010; Barquero et al., 2014). In addition, anatomical and physiological differences between the brains of children with dyslexia and of typically developing children can contribute to the classification of the former into subtypes that may respond to different forms of prevention and treatment (Galaburda, 2010). Moreover, recent work using eye-tracking technology has showed that the adoption of e-readers can support students with dyslexia in text comprehension (Zorzi et al., 2012; Schneps et al., 2013). Neural markers of dyscalculia are also starting to emerge together with new interventions that aim to strengthen numerosity processing, such as adaptive software (Butterworth et al., 2011). Neuroscientific findings further inform interventions in more severe neurodevelopmental disorders, such as Down syndrome. Individuals with Down syndrome present with a relative strength in visuospatial processing, which is coupled with a “striking preservation” of parietal and occipital gray matter (Pinter et al., 2001). At the same time, deficits are described in verbal processing and explicit memory (Fidler and Nadel, 2007). Thus, instruction that is presented with visual supports rather than a verbally-based instruction would be beneficial for children with Down syndrome.

### Scope of the present study

It has been recently shown that Greek teachers believe in the neuromyths that are also adopted by teachers elsewhere in the world (Deligiannidi and Howard-Jones, 2015). However, the teachers surveyed had a mean of teaching experience of 15.1 years, which translates into teachers being mostly in their late 30 s, a generation away from the internet generation currently studying at University. Therefore, it is important to extend current findings on Greek in-service teachers to students in Departments of Education, as it can be argued that they are more influenced by the nowadays widespread use of the internet compared to in-service teachers. A better understanding of currently in training, prospective teachers' needs is critical towards developing better teacher training programs.

The present study aims to study neuroscience literacy amongst prospective teachers in Greece, including their knowledge on the neuroscience of special education. It moreover aims to investigate the penetration of neuromyths amongst prospective teachers and identify factors that predict the adoption of neuromyths. Other goals include the investigation of whether prospective teachers are interested in gaining knowledge about the brain in the course of their studies and if they would like neuroscience courses to be included in their training curriculum. Students enrolled in two large universities were surveyed, namely the National and Kapodistrian University of Athens (NKUA) and the University of Thessaly (UoT). The former is located in the capital of Greece, while the latter is located in a smaller city, Volos.

The present study is primarily a survey, aiming at reporting descriptive findings. However, we also aimed to statistically test possible differences between undergraduate and graduate students as well as between students from the two universities. More specifically:

- Neuromyths: We hypothesized that undergraduate and postgraduate students will not differ in terms of their adoption of neuromyths nor will they differ in terms of their knowledge about the brain, as both groups will be surveyed before they have had the chance to attend the courses on neuroscience that are part of their curriculum. Students from the NKUA and the UoT are not expected to differ in terms of their adoption of neuromyths, following the same rationale.
- General knowledge about the brain: As above.
- Special education: We expect students from the UoT to have lower error scores when it comes to questions that target special education, as the majority of them attend a Department of Special Education. We do not expect to find any differences between undergraduate and graduate students, for the reasons described above.

With regards to possible predictors or neuromyths, we refrained form formulating a concrete hypothesis, as the literature in this topic has produced conflicting findings to date (Herculano-Houzel, 2002; Howard-Jones et al., 2009; Dekker et al., 2012; Gleichgerrcht et al., 2015).

## Materials and methods

### Participants

Five hundred seventy-three (516 female) undergraduate and postgraduate students enrolled in Departments of Education in two large universities in Greece, the NKUA (*n* = 336, mean age = 22.51 years, *SD* = 5.37, range = 20–52), and the UoT, (*n* = 218, mean age = 18.63 years, *SD* = 2.09, range = 18–37), participated in the study. The overwhelming proportion of women was expected as it reflects the typical sex ratio at Departments of Education; however, it did not allow for sex to be included as a variable in any of the analyses. The number of undergraduate students was 479 (mean age = 19.60 years, *SD* = 2.29, range = 18–44), and the number of postgraduate students was 94, (mean age = 26.99 years, *SD* = 7.16, range = 23–52). More specifically, students in five different programs of study were surveyed, (1) 257 undergraduate students from the Department of Primary Education at NKUA (232 female, mean age = 20.45 years, *SD* = 2.11, range = 20–44), (2) 117 undergraduate students from the Department of Special Education at UoT (104 female, mean age = 18.74 years, *SD* = 2.15, range = 18–31), (3) 105 undergraduate students from the Department of Preschool Education, at UoT (101 female, mean age = 18.50 years, *SD* = 2.02, range = 18–37), 78 postgraduate students from the Department of Primary Education at NKUA reading for the MSc in Special Education and Speech Therapy (66 female, mean age = 27.26 years, *SD* = 5.69, range = 23–47), and 16 postgraduate students from the Department of Primary Education at NKUA reading for the MSc in Sociobiology, Neuroscience and Education (13 female, mean age = 35.00 years, *SD* = 10.21, range = 23–52). The age difference between the undergraduate and the graduate students was significant as expected, *t*_(552)_ = −21.66, *p* < 0.001, and so was the age difference between students from the two universities, *t*_(552)_ = 10.20, *p* < 0.001, reflecting the fact that only in the NKUA were postgraduates surveyed. The study was carried out in accordance with the Declaration of Helsinki. All participation was voluntary; participants gave written informed consent and were debriefed after participation. The survey was anonymous and did not require an ethical approval.

### Instrument

A questionnaire was administered to all participants. The first part included 70 statements. Twenty-two statements were educational neuromyths (taken from Lilienfeld et al., 2011; Dekker et al., 2012), for example “We only use 10% of our brain.” The rest of the statements were general assertions about the brain (taken from Herculano-Houzel, 2002; Lilienfeld et al., 2011; Dekker et al., 2012; seven items developed by the present authors), for example “The brains of boys and girls develop at the same rate.” All seven items developed by the present authors were pertinent to special education, for example “Individuals with learning disabilities have a smaller brain,” “Boys are about 10 times more likely to be dyslexic compared to girls,” “Almost all autistic children are savants.” Two more items on special education were to be found in the original pool of items, namely “Learning problems associated with developmental differences in brain function cannot be remediated by education” (Dekker et al., 2012), “The brain of children with attention-deficit hyperactivity disorder (ADHD) are over-aroused” (Lilienfeld et al., 2011). The 63 items that were taken from previous questionnaires allow for a comparison between the present findings and findings from other studies and countries, whereas the seven newly-introduced items serve to expand the research field toward special education. None of the seven new items were treated as neuromyths, as their adoption or not by teachers has not been validated by previous research.

The presentation order of the myth and knowledge assertions was randomized, with the exception of the first 32 statements that were presented in the same order as the one used by Dekker et al. (2012) –which were randomized by those authors. Answering options for the 70 statements were “incorrect,” “correct,” and “do not know.” The “do not know” option was utilized to minimize accidental selection of the correct answer. Correct and incorrect assertions were balanced. The 70 items of the first part of the questionnaire are presented in the Appendix (Supplementary Material).

The second part of the questionnaire comprised of questions about the participants' personal interest in neuroscience, namely “Do you think it is useful for the teachers' educational practice to know how the brain works?” and “Do you think there should be a course on brain functions in the curriculum of Education Departments,” with possible responses being “Yes,” “No,” and “Perhaps.” Participants who answered “Yes” to the latter question were further asked “Do you think this course should be compulsory or optional,” with “compulsory” and “optional” being the two possible responses. Moreover, participants were asked how they draw relevant information themselves using the questions “Do you read magazines/newspapers or books on popular science topics” (response options: “Yes,” “Often,” “Rarely,” “No”) and “How many books (of any topic) do you read in a month?” (response options being 1, ½, 1, 2, 3, 4, more). Finally, there were questions about the teachers knowledge of “brain-based” educational approaches (e.g., BrainGym^©^) (response options being “Yes” and “No”). Information about the participants' sex, education, and age was also collected.

### Procedure

The questionnaire was administered during the first lecture of modules on neuroscience, where such myths were later debunked. Participation was voluntary and the completion of the questionnaire lasted about 20 minutes. The term neuromyth was mentioned only after the questionnaires had been completed and collected.

### Statistical analysis

The data was analyzed using the Statistical Package for the Social Sciences (SPSS), version 23.0 for Mac. Error scores for neuromyths and general assertions were calculated by taking into account only the incorrect responses made by the participants (and not the “do not know” answers), following common practice in the field of neuromyths (see Ferrero et al., 2016, for a meta-analysis of findings); the scores were transformed into percentages. The nine items that are pertinent to special education were grouped together (items 28, 34, 49, 54, 56, 58, 61, 64, 66) for further analysis using the sum of the error scores. Normality of data distribution was assessed using the two-sample Shapiro-Wilk test, with the two universities (NKUA and UoA) and the status of the students (undergraduate or graduate) as the two groups. Since the error scores of the neuromyths, the general assertions about the brain, and the special education category were found not to follow a normal distribution (all *p* < 0.001), the non-parametric Mann-Whitney *U*-test was employed to examine differences in average ranks in the error scores between the two universities (NKUA and UoA) and the status of the students (undergraduate or graduate). Interactions of university with student status could not be calculated, as only undergraduate and not postgraduate students from UoT participated in the study, whereas both undergraduate and postgraduate students were surveyed in the NKUA. Moreover, chi-square (χ^2^) analyses were performed on categorical items.

To examine factors predicting neuromyths, a regression analysis was performed for percentage of error scores for neuromyths (dependent variable) and status, university, reading of popular science, number of books read every month and error score of general assertions about the brain as predictors. All analyses used a α = 0.05 level for statistical significance.

## Results

### Neuromyths

Table 1 presents the responses made for each neuromyth. The mean error score was 43.62% (*SD* = 10.96). No differences were found between postgraduate and graduate students, *U* = 22.33, *p* = 0.95, but differences were found between students of the two universities (NKUA and UoT), *U* = 33.83, *p* = 0.01, with NKUA students having a higher mean rank (*M* = 299.82) than UoT students (*M* = 264.12). A lot of variation was found between myths. Eight of the 22 myths were answered incorrectly from at least 50% of the participants. The most prevalent of these myths were [1] “Individuals learn better when they receive information in their preferred learning style (e.g., auditory, visual, kinesthetic)” (94.4% of the participants erroneously believed that this is correct), [2] “Environments that are rich in stimuli improve the brains of pre-school children” (90.3% of the participants erroneously believed this is correct), and [3] “Exercises that rehearse co-ordination of motor-perception skills can improve literacy skills” (77.5% of the participants erroneously believed this is correct). On the other hand, some myths were answered correctly by most of the participants, for example “Individual learners show preferences for the mode in which they receive information (e.g., visual, auditory, kinesthetic)” (93.9% of the participants correctly believed this is true) and “Raising children similarly leads to similarities in their adult personalities” (89.4% of the participants correctly believed that this assertion is untrue).

**Table 1:** **Correctness of responses for all myth assertions**.

**Neuromyth**	**Correct (%)**	**Incorrect (%)**	**Do not know (%)**	***n***
Children must acquire their native language before a second language is learned. If they do not do so neither language will be fully acquired. (D)	35.8	55.6	8.6	570
If pupils do not drink sufficient amounts of water (6–8 glasses a day) their brains shrink. (D)	46.1	8.6	45.3	573
We only use 10% of our brain. (D, H, L)	25.7	47.4	26.9	572
Differences in hemispheric dominance (left brain, right brain) can help explain individual differences amongst learners. (D, L)	7.9	55	37.1	573
There are critical periods in childhood after which certain things can no longer be learned. (D)	31.2	48.1	20.7	570
Individuals learn better when they receive information in their preferred learning style (e.g., auditory, visual, kinesthetic). (D)	3.7	94.4	1.9	571
Regular drinking of caffeinated drinks reduces alertness. (D)	31.9	41	27.1	571
Exercises that rehearse co-ordination of motor-perception skills can improve literacy skills. (D)	4.2	77.6	18.2	570
Extended rehearsal of some mental processes can change the shape and structure of some parts of the brain. (D)	31.1	27.5	41.4	570
Individual learners show preferences for the mode in which they receive information (e.g., visual, auditory, kinesthetic). (D, L)	93.9	2.8	3.3	571
Learning problems associated with developmental differences in brain function cannot be remediated by education. (D)	55.5	24.2	20.3	571
Short bouts of coordination exercises can improve integration of left and right hemispheric brain function. (D)	7.8	36.7	55.5	566
The brain of children with attention-deficit hyperactivity disorder (ADHD) are over-aroused. (L)	10.8	40	49.2	572
IQ scores are unrelated to school performance. (L)	42.2	52.4	5.4	569
Raising children similarly leads to similarities in their adult personalities. (L)	89.4	7.9	2.7	573
Visual perceptions are accompanied by tiny emissions from the eyes. (L)	20	25.8	54.2	570
Human memory works like a tape recorder or video camera and accurately records the events we've experienced. (L)	43.6	44.7	11.7	573
Individuals can learn new information, like new languages, when asleep. (L)	38.7	38.2	23.1	571
It has been scientifically proven that fatty acid supplements (omega-3 and omega-6) have a positive effect on academic achievement. (D)	8.6	53.6	37.8	573
Environments that are rich in stimuli improve the brains of pre-school children. (D)	3.9	90.3	5.8	568
Our handwriting reveals our personality. (L)	15.7	71.7	12.6	572
IQ scores almost never change over time. (L)	61.6	20.6	17.8	573

*The percentages listed above how the percentage of participants that responded to these statements correctly, incorrectly, or whether they did not know. For example, 94.4% of the participants claimed that the assertion “Individual learners show preferences for the mode in which they receive information (e.g., visual, auditory, kinesthetic)” is true, even though this is a false statement, therefore they have responded incorrectly*.

*D, item taken from Dekker et al. (2012)*.

*H, item taken from Herculano-Houzel (2002)*.

*L, item taken from Lilienfeld et al. (2011)*.

### General knowledge of the brain

With respect to the general assertions about the brain (see Table 2) the mean error score was 21.06% (*SD* = 7.99). No differences were found between postgraduate and graduate students *U* = 24.87, *p* = 0.06, or between students of the two universities (NKUA and UoT), *U* = 38.35, *p* = 0.98. Three statements were answered correctly by 89% of the participants or more. These were (a) “Mental capacity is hereditary and cannot be changed by the environment or experience” (89.3% correctly believed this is untrue), (b) “There are sensitive periods in childhood when it's easier to learn things (89.3% correctly believed this is true),” and (c) “When we sleep, the brain shuts down” (92.3% correctly believed this to be untrue). The assertion that received the highest percentage of incorrect responses was “Any brain region can perform any function” (78.1% erroneously believed this is correct).

**Table 2:** **Correctness of responses for general statements**.

**General statements**	**Correct (%)**	**Incorrect (%)**	**Don't know (%)**	***n***
We use our brains 24 h a day. (D, H)	76.9	20.5	2.6	572
Boys have bigger brains than girls. (D)	9.5	56.8	33.7	570
When a brain region is damaged other parts of the brain can take up its function. (D, H)	14.1	59.9	26	573
The left and right hemisphere of the brain always work together. (D)	12.4	57.5	30.1	572
The brains of boys and girls develop at the same rate. (D	56.1	20.1	23.8	572
Brain development has finished by the time children reach secondary school. (D)	54.2	17.8	28	572
Information is stored in the brain in a network of cells distributed throughout the brain. (D, H)	46.2	18.5	35.3	573
Learning is not due to the addition of new cells to the brain. (D)	50.5	18.9	30.6	571
Learning occurs through modification of the brains' neural connections. (D, H)	46.3	8.8	44.9	570
Academic achievement can be affected by skipping breakfast. (D)	76	10.7	13.3	570
Normal development of the human brain involves the birth and death of brain cells. (D, H)	60.3	11.2	28.5	569
Mental capacity is hereditary and cannot be changed by the environment or experience. (D, H)	89.3	6.8	3.9	570
Vigorous exercise can improve mental function. (D)	62.3	12.5	25.2	570
Children are less attentive after consuming sugary drinks and/or snacks. (D)	33.3	33.1	33.6	571
Circadian rhythms (“body-clock”) shift during adolescence, causing pupils to be tired during the first lessons of the school day. (D)	34.7	13	52.3	571
Production of new connections in the brain can continue into old age. (D)	29.5	27.9	42.6	570
There are sensitive periods in childhood when it's easier to learn things. (D)	89.3	2.6	8.1	570
When we sleep, the brain shuts down. (D)	92.3	4.9	2.8	573
The brain is the body organ that consumes the most oxygen relative to its size. (H)	61.8	3.5	34.7	573
Communication between different parts of the brain happens through electrical impulses and chemical substances. (H)	51.7	9.1	39.2	572
Tobacco's nicotine has a direct effect on the brain. (H)	75.3	7.5	17.2	571
It is with the brain, and not with the heart, that we experience happiness, anger, or fear. (H)	70.6	17.8	11.6	572
To learn how to do something, it is necessary to pay attention to it. (H)	77.4	17.9	4.7	571
Performance in activities such as playing the piano improves as a direct function of the number of hours spent practicing. (H)	60	22.3	17.7	573
Mental effort does not raise oxygen consumption by the brain. (H)	44.6	13.6	41.8	572
Knowing our brain we can understand better how our thoughts, our reasoning, and our memories work. (H)	73.6	9.2	17.2	573
Body function regulation through hunger, thirst, and temperature control are functions of a certain brain area. (H)	61.2	9.4	29.4	572
The brain itself is not sensitive to pain; this is why brain surgery can be performed under local anesthesia. (H)	15.6	38.6	45.8	570
In the majority of right-handed people, speech is a specialty of the left brain hemisphere. (H)	59.3	5.9	34.8	573
An epileptic crisis results from the temporary silencing of a brain area; this is why epileptics lose consciousness during a crisis. (H)	8	52.1	39.9	572
Brain activity can be studied through the oxygen consumption of specific brain areas. (H)	33.6	8.4	58	572
The enhancement of the sense of touch in the blind is due to an increase in the number of receptors in the fingertips and not to changes in the brain. (H)	38.7	24	37.3	571
Our brain has maps of the surface of the body and of the visual field. (H)	38.2	7	54.8	570
Dyslexia is associated with intelligence. (O)	68.6	18.7	12.7	573
The electrical activity of the brain of a dreaming person is similar to that of a waking person. (H)	21.4	40.4	38.2	569
Boys are about 10 times more likely to be dyslexic compared to girls. (O)	9.5	44.7	45.8	570
Any brain region can perform any function. (H)	78.1	7.9	14	571
Locomotion consists of a series of reflexes; this is why we can do other things and walk at the same time. (H)	77.6	5.1	17.3	572
Almost all autistic children are savants. (O)	17.9	27.1	55	571
Varied sensory experience is necessary to the normal maturation of the brain functions. (H)	78.1	3.3	18.6	571
Left-handed individuals don't have a higher IQ than right-handed individuals. (O)	38.6	34.5	26.9	572
The defining feature of dyslexia is reversing letters. (O)	21.6	62.1	16.3	573
Dreaming doesn't occur any time during sleep. (H)	63.3	17.3	19.4	573
Individuals with learning disabilities have a smaller brain. (O)	79.6	2.1	18.3	573
Being right- or left-handed is a matter of being, respectively, left or right brain hemisphere dominant. (H)	9.4	65.1	25.5	573
The brain has areas specialized at certain functions, such as mathematics; the development of these brain areas can be identified through the shape of the skull. (H)	23.3	21.1	55.6	572
Without a brain, consciousness is not possible. (H)	78.1	3.8	18.1	570
The bigger the brain, the more intelligent the animal. (H)	56.8	13	30.2	569

*D, item taken from Dekker et al. (2012)*.

*H, item taken from Herculano-Houzel (2002)*.

*L, item taken from Lilienfeld et al. (2011)*.

*O, item developed by the authors*.

### Special education

The nine questions that were pertinent to special education were further analyzed in isolation. The mean error score was 36.86% (*SD* = 20.38). Mann-Whitney *U* tests were performed with status of students (graduate or undergraduate) and university (NKUA or UoT) as the between-participant variables and the error score in the special education category as the dependent variable. No significant difference was found between undergraduates and graduates (*p* = 0.99), but a significant difference was found between students of the two universities, with the NKUA students having a higher mean rank (*M* = 307.24) than UoT students (*M* = 252.37). The statement with the highest correct score in this category was “Individuals with learning disabilities have a smaller brain” (79.6% correctly believed this is not true), the item with the highest incorrect score was “The defining feature of dyslexia is reversing letters” (62.1% wrongly believed it is true), and the item with the highest “don't know” score was “Almost all autistic children are savants” (55% responded that they did not know the answer.)

### General questions

The responses to the rest of the questionnaire items (e.g., participants' interest in neuroscience) were also analyzed (see Supplementary Table 1). The question “Do you think it is useful for the teachers' educational practice to know how the brain works?” was answered positively by 88.4% of the students, with 2.4% answering negatively and 9.2% answering perhaps. Chi-square analyses showed that there was not a significant difference in responses between either undergraduate or postgraduate students and between students of the two universities (all *ps* > 0.47). The question “Do you think there should be a course on brain functions in the curriculum of Education Departments?” was answered positively by 83.6% of the students, with 3.4% answering negatively and 13% answering perhaps. Chi-square analyses showed that there was no significant difference in responses between either undergraduate or postgraduate students and between students of the two universities (all *ps* > 0.14). Of those who answered positively to the previous question, 65.3% thought that such a course should be compulsory and 34.7% that it should be optional, with 70.86% of the students in the NKUA believing that it should be mandatory compared to 56.63% in the UoT (χ^2^ = 10.61, *p* = 0.001). No difference was found between undergraduate and graduate students (*p* = 0.21). Applications such as BrainGym© were known to 19.0% of the sample, with no statistically significant differences between undergraduate and postgraduate students and between students in the two universities (all *p*s > 0.12).

### Predictors of neuromyths

A regression was run in order to investigate predictors of neuromyths with percentage of error scores for neuromyths as the dependent variable and student status, university, reading of popular science, number of books read every month and percentage of error scores on general assertions as predictors. The model (see Table 3) significantly explained a small proportion of the variance (*R*^2^ = 0.07) in myth scores, *F*_(5, 529)_ = 7.21, *p* < 0.01. The error score for neuromyth assertions was significantly predicted by the error score for general knowledge about the brain (β = 0.34, *p* < 0.001). This indicates that participants with higher error scores on general knowledge assertions were more likely to believe in myths. None of the other factors (graduate status, university, reading of popular science, or number of books read every month) predicted answering wrongly in myth statements.

**Table 3:** **Predictors of neuromyths error score**.

	**B (SE)**	***t***	***p***
Constant	42.21 (3.45)	12.20	0.000
Graduate status	−2.02 (1.39)	−1.46	0.15
University	−1.22 (1.05)	−1.16	0.25
Number of books read every month	−0.53 (0.34)	−1.55	0.12
Reading of popular science	−1.00 (0.56)	−1.80	0.07
Error score on general assertions	0.34 (0.06)	5.59	0.000

## Discussion

The present study examined the prevalence of neuromyths, as well as the neuroscience literacy among undergraduate and postgraduate students in Departments of Education in two Greek universities, the National and Kapodistrian University of Athens (NKUA) and the University of Thessaly (UoT). Factors related to these outcomes were also surveyed. Findings showed a worrying penetration of neuromyths amongst prospective teachers, while neuroscience literacy was fairly good. General knowledge about the brain was the only significant predictor of neuromyths. Prospective teachers believed it is important to acquire knowledge of neuroscience and that courses on brain function should be part of the curriculum of Education Departments.

### Findings on neuromyths and brain knowledge

With regards to general knowledge about the brain, the error score over the whole sample was 21.06%, with no significant differences between undergraduate and postgraduate students or between students of the two universities. Nearly 90% of the participants acknowledged that environment or experience can affect mental capacity—a belief that can only act as a valuable tool in a teacher's pursuit to develop the capacities of her students. On the other hand, the assertion that was answered incorrectly by about 78.1% of prospective teachers was that “Any brain region can perform any function.” Of note, prospective teachers were surveyed before they had had the chance to attend the courses on neuroscience that were part of their curriculum, so these figures represent general knowledge acquired by other means, such as school education, reading books, or popular science.

Prospective teachers were found to believe in a mean 43.62% of the neuromyths presented, while eight out of the 20 myths were answered incorrectly by at least 50% of the participants. These figures are better than those reported in Gleichgerrcht et al. (2015), where more than 50% of teachers in Latin America failed to identify 9 of the 12 invalid statements as such, and slightly better than those reported in Dekker et al. (2012), where British and Dutch teachers believed in more than half of the myths. They are also in line with the figures reported in samples of Greek (Deligiannidi and Howard-Jones, 2015), Turkish (Karakus et al., 2015), and Chinese teachers (Pei et al., 2015). A significant difference with regards to the adoption of neuromyths was found between the two universities, with students from the NKUA having higher error scores compared to students from the UoT. However, the actual difference in error scores was less than 3%, which makes this difference not meaningful. With large sample sizes like the one in the present study, even small differences can be statistically significant.

The most prevalent myth was the belief in learning styles, in other words that “Individuals learn better when they receive information in their preferred learning style (e.g., auditory, visual, kinaesthetic),” which was embraced by a staggering 94.4% of our sample. Together with learning styles, the triad of most popular neuromyths comprised of “Environments that are rich in stimuli improve the brains of pre-school children” (90.3%) and “Exercises that rehearse co-ordination of motor-perception skills can improve literacy skills” (77.5%). Our findings are in line with current literature on neuromyth adoption. For example, Deligiannidi and Howard-Jones (2015) in their sample of Greek teachers found that 97% of their sample endorsed both the learning styles and the rich environments myth, while Karakus et al. (2015) found that 97.1% of their sample of Turkish teachers adopted the learning styles myth. In a sample of 44 in-service high-school teachers, 57 college teachers and 160 first year primary student teachers from the French-speaking part of Switzerland, more than 87% believed that a pedagogical approach based on a learning types distinction favors learning (Tardif et al., 2015). Teachers in East China also appear to adopt these myths in similar percentages, for example 97% adopt the learning styles myth, 89% the rich environments myth and 79% the exercises myth (Pei et al., 2015). A recent meta-analysis by Ferrero et al. (2016) has indeed showed that the learning style and the rich environment myths are extraordinarily popular in most countries. However, differences are also to be found. For example, our sample was more enthusiastic about attention (77.4% believe “To learn how to do something, it is necessary to pay attention to it”), compared to East China teachers (40%). Moreover, 24.2% of our sample and 16% of the UK sample believe that “Learning problems associated with developmental differences in brain function cannot be remediated by education,” whereas this figure was 50% for the East China teachers (Dekker et al., 2012; Pei et al., 2015), 11.2% for Peruvian teachers, 5.6% for teachers from Argentina, and 6% for teachers from Chile (Gleichgerrcht et al., 2015).

Items that are pertinent to special education were isolated for further analysis. Neuroscientific knowledge on special education issues has not been investigated before. It was found that the mean error score for the items that were pertinent to special education (36.86%) was higher than the mean error score for the general assertions about the brain in total (21.06%). This finding demonstrates that prospective teachers are not as knowledgeable with regards to the neural underpinnings of disorders that require special education as they are with regards to the typical brain. Moreover, it was found that the students in the NKUA had higher error scores compared to the UoT. The UoT includes a Department of Special Education, so it can be inferred that the student who enroll in this department are individuals who have been self-selected for their interest in special education and have individually sought more information in issues relating to it.

### Findings on predictors of neuromyths

A regression analysis revealed that the brain knowledge error score was the only statistically significant predictor of the neuromyths error score. In other words, general knowledge about the brain acted as a protective shield against believing in neuromyths. This finding is in line with previous findings of Howard-Jones et al. (2009), but in contrast to Dekker et al. (2012) and Gleichgerrcht et al. (2015), who found that individuals who reported knowing more about the brain were more likely to believe in neuromyths. One possible explanation for this discrepancy in findings could be the fact that Dekker et al. (2012) and Gleichgerrcht et al. (2015) both surveyed in-service teachers. On the other hand, the findings from Howard-Jones et al. (2009) as well as the present study come from trainee teachers. According to Dekker et al. (2012), in-service teachers, especially those ones who are eager to implement neuroscience findings in their teaching, might have been confronted by more information compared to trainee teachers about the brain, both correct and incorrect. It could be the case that they are then unable to differentiate between the two. The eagerness to implement findings from the field of educational neuroscience coupled with the lack of expertise in the field might make them more vulnerable to the adoption of neuromyths, while at the same time also making them more knowledgeable about the brain. Ferrero et al. (2016) proposed that the fact that responders who seem to know more about the brain are also more susceptible to adopting neuromyths might also be explained by an acquiescence bias, in other words, teachers who tend to respond affirmatively to a greater number of general assertions about the brain, also give more affirmative assertions to neuromyths items. However, this cannot be the case in the present study, as the items are phrase in such a way that half of the statements were correct and the other half incorrect.

### Future directions and suggestions

In light of the above findings, and in order to reduce the number misconceptions that currently proliferate within schools, we suggest enhancing the neuroscience literacy of prospective teachers by incorporating neuroscience courses into their initial teacher education. In doing so, we align with a number of other authors and organizations who have suggested including neuroscience in undergraduate teacher education and professional development (e.g., Ansari and Coch, 2006; Goswami, 2006; Pickering and Howard-Jones, 2007; Lindell and Kidd, 2011; Royal Society, 2011; Dubinsky et al., 2013; Rato et al., 2013; Busso and Pollack, 2015; Tardif et al., 2015). Such instruction can improve the quality of teaching, a rather critical point, as evidence shows that teacher quality is a significant predictor of children's educational success (Organisation for Economic Co-operation Development, 2004). As shown by our findings and as recommended by the Royal Society (2011), training and continued professional development should include a component of neuroscience relevant to special education. Future studies should evaluate changes in prospective teachers' neuroscience literacy and belief in neuromyths after taking courses on neuroscience in order to provide evidence for their effectiveness and to further quantify their effect.

While acknowledging that every new learning module might need to replace an existing one in order to keep the workload of the students constant, we believe that education needs to evolve over time and incorporate new relevant knowledge and expertise. Psychology (cognitive, educational, social) is already an important part of teacher education. Cognitive, development and social neuroscience can expand the learning horizons of these disciplines and compliment them in adding an extra (neurobiological) level of explanation (Diamond and Amso, 2008), thereby deepening the understanding of teachers regarding the learning brain (Hille, 2011). Neuroscience should aim to assist prospective teachers develop a better understanding of topics relevant to education, and not be used as a prescriptive tool (Ansari and Coch, 2006). At the same time, continuing professional development should include neuroscience-based short courses, seminars, or workshops, as practicing teachers are not likely to have benefited from such input during their training years.

Teacher education on neuroscience should be developed on the basis of a bidirectional dialogue between researchers and practitioners, in order for topics relevant to multiple learning situations to be identified and covered, but also for neuroscience to be translated into a language shared by teachers. The need of a greater communication between neuroscientists and teachers has in fact been highlighted by a number of researchers (e.g., Goswami, 2004; Ansari and Coch, 2006; Fisher et al., 2010; Lindell and Kidd, 2011; Rato et al., 2013; Tardif et al., 2015), and has been further recommended by the Royal Society (2011). Goswami (2006) has suggested that this communication might be better mediated by research communicators, as they might be better placed to interpret and communicate neuroscience findings in the language of educators, and to feedback questions, ideas, and criticisms to the neuroscientists. Teachers do acknowledge the importance of knowledge about the brain in directing their teaching to best practices and also help protect them from unscientific claims and they are, therefore, eager to acquire brain knowledge and be informed about related research that could impact their teaching (Fang, 1996; MacNabb et al., 2006; Pickering and Howard-Jones, 2007; Zambo and Zambo, 2009, 2011; Bartoszeck and Bartoszeck, 2012; Serpati and Loughan, 2012; Rato et al., 2013; Karakus et al., 2015). For example, in Karakus et al. (2015) 93.2% of the 259 participants was supportive of the idea that neuroscience is relevant to education and 90.3% expressed a desire to acquire more knowledge about the brain. Serpati and Loughan (2012) reported that 94% of the 221 teachers participating in their study agreed that it is significant to acquire knowledge on the neurological underpinnings of learning, cognition, and behavior, while 73% of the 170 Minnesota fifth- through eighth-grade teachers surveyed by mail by MacNabb et al. (2006) stated that they saw a need for training of life science teachers in neuroscience and 80% reported that they would benefit from this kind of training. The support of teachers is critical, as teachers' beliefs is the most important factor in determining the success or failure of a new teaching approach (Errington, 2004).

Other suggestions for promoting brain knowledge amongst teachers include a regular, interactive e-mail/online “digest” of Neuroscience Education News, presenting an accurate summary of the latest findings relevant to the teacher in the classroom (Lindell and Kidd, 2011). Moreover, teachers can be introduced and directed to web sites with valid information on the brain, for example Brain Connection (http://www.brainconnection.com), the Dana Alliance for Brain Initiatives (http://www.dana.org/braincenter), and Teach the Brain (Goswami, 2004; Zambo and Zambo, 2009). These suggestions have not been tested to date for their effectiveness; therefore future studies might need to do so.

It is important to note that findings by Weisberg et al. (2008) show that individuals that have attended introductory cognitive neuroscience classes were misled by bogus neuroscientific explanations in the same way as laypeople. It was only individuals that can be considered experts, as they were pursuing or had a degree in cognitive neuroscience or related areas, who could identify such nonsense neuroscientific explanations. Given the usual time constraints, student teachers cannot be expected to become experts. So, introductory modules should emphasize on debunking well-known neuromyths as well as on training the students to critically consume neuroscientific findings. Research on myths in the field of psychology has indeed shown that one of the most effective, evidence-based way to confront scientific myths is to directly refute misconception in introductory classes (Guzzetti et al., 1993; Kowalski and Taylor, 2009, 2011). Developing an understanding of how research is conducted and presented in neuroscience should take priority over fact-learning, which will possibly be outdated in a few years time (Ansari and Coch, 2006). Neuroscience is a field constantly developing and changing, thus teachers should be equipped to follow the new developments, by effectively reading and critically evaluating the information they are bound to receive from multiple sources. When it comes to brain-based products, Sylvan and Christodoulou (2010) have published a guide to educators to help them make informed decision, which comprises of five steps that educators should take, namely identifying educational goals and populations, aligning goals and product purposes, reviewing product merits, identifying the product's benefits and limitations, and characterizing the product's impact on behavioral performance. Such guides could also be part of introductory courses.

## Conclusion

In conclusion, the present research showed that prospective teachers in Greece are enthusiastic about improving their neuroscience literacy. Like teachers from other countries, they do adopt neuromyths, but they also seem to have a basic knowledge about the brain. Findings further showed that general knowledge is the best safeguard against believing in neuromyths. The present results also showed that prospective special education teachers can benefit from academic instruction on neuroscience. We suggest that such instruction takes place in undergraduate courses of Departments of Education and that emphasis is given in debunking neuromyths, enhancing critical reading skills, and dealing with topics relevant to special education.

## Ethics statement

The study was carried out in accordance with the recommendations of the Helsinki declaration and written consent was secured. Participation was voluntary and required only the completion of a questionnaire where participants had to respond anonymously with a “true,” “false,” or “don't know” response to various statements about the brain.

## Author contributions

MPP contributed to the conception and design of the work; the acquisition, analysis, and interpretation of the data; and drafted the manuscript. EH contributed to the analysis of the work and to manuscript revision. FV contributed to the design of the work; the acquisition, and interpretation of the data; and to manuscript revision. All authors approved the final version of the manuscript.

### Conflict of interest statement

The authors declare that the research was conducted in the absence of any commercial or financial relationships that could be construed as a potential conflict of interest.
