# Supplementary material for: Brain Knowledge and the Prevalence of Neuromyths among Prospective Teachers in Greece
Source: Front Psychol. 2017 May 29;8:804. doi: 10.3389/fpsyg.2017.00804 (PMC5447089; doi:10.3389/fpsyg.2017.00804)
Supplement: Supplementary file 1 [file Table1.pdf]

Table 1 – Supplementary material
Aggregated data on students' views on the rest of the questionnaire items.

	Department of Primary Education. University of Athens (undergraduate) (*n* = 257)	Department of Special Education. University of Thessaly (undergraduate) (*n* = 117)	Department of Preschool Education. University of Thessaly (undergraduate) (*n* = 105)	Department of Primary Education. University of Athens (postgraduate Program "Special Education and Speech Therapy") (*n* = 78)	Department of Primary Education. University of Athens (postgraduate program "Sociobiology, Neuroscience, and Education") (*n* = 16)	Total sample
"Do you think it is useful for the teachers' educational practice to know how the brain works?"	90.3% Yes<br>13% No<br>8.4% Perhaps	90.5% Yes<br>1.7% No<br>7.8% Perhaps	82.8% Yes<br>4.8% No<br>12.4% Perhaps	84.7% Yes 3.8% No<br>11.5% Perhaps	100% Yes	88.4% Yes 2.4% No<br>9.2% Perhaps
"Do you think there should be a course on brain functions in the curriculum of the Education Departments?"	86.9% Yes<br>1.3% No<br>11.8% Perhaps	86.2% Yes<br>3.4% No<br>10.4% Perhaps	73.3% Yes<br>6.4% No<br>14.1% Perhaps	79.5% Yes 6.4% No<br>14.1% Perhaps	100% Yes	83.6% Yes 3.4% No<br>13% Perhaps

"If you answered YES to the previous question, do you think that this course should be: Compulsory or Optional?"	75.5% Compulsory<br>24.5% Optional	54.5% Compulsory<br>45.5% Optional	59.3% Compulsory<br>40.7% Optional	54.3% Compulsory<br>45.7% Optional	81.3% Compulsory<br>18.7% Optional	65.3% Compulsory<br>34.7% Optional
"Do you read magazines / newspapers or books on popular science topics?"	9.6% Yes<br>13.8% Often<br>59.4% Rarely 17.2% No	11.2% Yes<br>15.6% Often<br>42.2% Rarely<br>31% No	7.7% Yes<br>6.7% Often<br>53.9% Rarely<br>31.7% No	17.1% Yes<br>31.6% Often<br>38.1% Rarely<br>13.2% No	25% Yes<br>18.7% Often<br>50% Rarely<br>6.3% No	11.1% Yes<br>15.4% Often<br>51.5% Rarely<br>22% No
"How many books (of any topic) do you read in a month?"	11.1%: 0<br>16.2%: ½<br>33.2%: 1<br>24.6%: 2<br>8.4%: 3<br>1%: 4<br>5.5%: More	13.5%: 0<br>16.2%: ½<br>31.6%: 1<br>18%: 2<br>10.8%: 3<br>4.5%: 4<br>5.4%: More	9.6%: 0<br>13.5%: ½<br>37.6%: 1<br>24%: 2<br>6.7%: 3<br>4.8%: 4<br>3.8%: More	3.8%: 0<br>14%: ½<br>38.5%: 1<br>28.2%: 2<br>10.3%: 3<br>2.6%: 4<br>2.6%: More	12.5%: 0<br>31.3%: ½<br>37.4%: 1<br>12.5%: 2<br>6.3%: 3<br>0%: 4<br>0%: More	9.8%: 0<br>15.3%: ½<br>34.4%: 1<br>24.1% :2<br>9%: 3<br>2.8%: 4<br>4.6%: More
"Are you familiar with educational applications based on neuroscience, as the Brain Gym;»	13.2% Yes<br>86.8% No	22.4% Yes<br>77.6% No	24.5% Yes<br>75.5% No	22.1% Yes<br>77.9% No	31.2% Yes<br>68.8% No	19% Yes<br>81% No

"Are you interested in scientific knowledge about the brain functions and how they affect the process of learning?"	78.9% Yes<br>5.2% No<br>15.9% Perhaps	78.3% Yes<br>4.3% No<br>17.4% Perhaps	73.6% Yes<br>8.8% No 17.6% Perhaps	79.2% Yes<br>5.2% No 15.6% Perhaps	100% Yes	78.4% Yes<br>5.5% No<br>16.1% Perhaps
