# Supplementary material for: Brain Knowledge and the Prevalence of Neuromyths among Prospective Teachers in Greece
Source: Front Psychol. 2017 May 29;8:804. doi: 10.3389/fpsyg.2017.00804 (PMC5447089; doi:10.3389/fpsyg.2017.00804)
Supplement: Supplementary file 2 [file DataSheet1.pdf]

Appendix

1. We use our brains 24h a day (C).
2. *Children must acquire their native language before a second language is learned. If they do not do so neither language will be fully acquired* (I).
3. Boys have bigger brains than girls (C).
4. *If pupils do not drink sufficient amounts of water (D6–8 glasses a day) their brains shrink (I).*
5. *It has been scientifically proven that fatty acid supplements (omega-3 and omega-6) have a positive effect on academic achievement (I).*
6. When a brain region is damaged other parts of the brain can take up its function (C).
7. *We only use 10% of our brain (I).*
8. The left and right hemisphere of the brain always work together (C).
9. *Differences in hemispheric dominance (left brain, right brain) can help explain individual differences amongst learners (I).*
10. The brains of boys and girls develop at the same rate (I).
11. Brain development has finished by the time children reach secondary school (I).
12. *There are critical periods in childhood after which certain things can no longer be learned (I).*
13. Information is stored in the brain in a network of cells distributed throughout the brain (C).
14. Learning is not due to the addition of new cells to the brain (C).
15. *Individuals learn better when they receive information in their preferred learning style (e.g., auditory, visual, kinesthetic) (I).*
16. Learning occurs through modification of the brains' neural connections (C).
17. Academic achievement can be affected by skipping breakfast (C).
18. Normal development of the human brain involves the birth and death of brain cells (C).
19. Mental capacity is hereditary and cannot be changed by the environment or experience (I).
20. Vigorous exercise can improve mental function (C).
21. *Environments that are rich in stimulus improve the brains of pre-school children* (I).
22. Children are less attentive after consuming sugary drinks and/or snacks (I).
23. Circadian rhythms ("body-clock") shift during adolescence, causing pupils to be tired during the first lessons of the school day (C).
24. *Regular drinking of caffeinated drinks reduces alertness(C).*
25. *Exercises that rehearse co-ordination of motor-perception skills can improve literacy skills (I).*
26. *Extended rehearsal of some mental processes can change the shape and structure of some parts of the brain (C).*
27. *Individual learners show preferences for the mode in which they receive information (e.g., visual, auditory, kinesthetic (C)).*
28. *Learning problems associated with developmental differences in brain function cannot be remediated by education (I).*
29. Production of new connections in the brain can continue into old age (C).
30. *Short bouts of coordination exercises can improve integration of left and right hemispheric brain function (I).*
31. There are sensitive periods in childhood when it's easier to learn things (C).
32. When we sleep, the brain shuts down (I).
33. The brain is the body organ that consumes the most oxygen relative to its size (C).
34. *The brain of children with attention-deficit hyperactivity disorder (ADHD) are over-aroused (I).*

35. Communication between different parts of the brain happens through electrical impulses and chemical substances (C).
36. Tobacco's nicotine has a direct effect on the brain (C).
37. *IQ scores are unrelated to school performance (I).*
38. It is with the brain, and not with the heart, that we experience happiness, anger, or fear (C).
39. To learn how to do something, it is necessary to pay attention to it (C).
40. *Raising children similarly leads to similarities in their adult personalities (I).*
41. Performance in activities such as playing the piano improves as a direct function of the number of hours spent practicing (C).
42. Mental effort does not raise oxygen consumption by the brain (I).
43. Knowing our brain we can understand better how our thoughts, our reasoning, and our memories work (C).
44. *Visual perceptions are accompanied by tiny emissions from the eyes (I).*
45. Body function regulation through hunger, thirst, and temperature control are functions of a certain brain area (C).
46. *Human memory works like a tape recorder or video camera, and accurately records the events we've experienced (I).*
47. The brain itself is not sensitive to pain; this is why brain surgery can be performed under local anesthesia (C).
48. In the majority of right-handed people, speech is a specialty of the left brain hemisphere (C).
49. An epileptic crisis results from the temporary silencing of a brain area; this is why epileptics lose consciousness during a crisis (I).
50. *Individuals cannot learn new information, like new languages, when asleep (C).*
51. *Our handwriting reveals our personality (I).*
52. Brain activity can be studied through the oxygen consumption of specific brain areas (C).
53. *IQ scores almost never change over time (I).*
54. The enhancement of the sense of touch in the blind is due to an increase in the number of receptors in the fingertips, and not to changes in the brain (I).
55. Our brain has maps of the surface of the body and of the visual field (C).
56. Dyslexia is associated with intelligence (I).
57. The electrical activity of the brain of a dreaming person is similar to that of a waking person (C).
58. Boys are about 10 times more likely to be dyslexic compared to girls (I).
59. Any brain region can perform any function (I).
60. Locomotion consists of a series of reflexes; this is why we can do other things and walk at the same time (C).
61. Almost all autistic children are savants (I).
62. Varied sensory experience is necessary to the normal maturation of the brain functions (C).
63. Left-handers don't have higher IQ scores than right-handers (C).
64. The defining feature of dyslexia is reversing letters (I).
65. Dreaming doesn't occur any time during sleep (C).
66. Individuals with learning disabilities have a smaller brain (I).
67. Being right- or left-handed is a matter of being, respectively, left or right brain hemisphere dominant (I).
68. The brain has areas specialized at certain functions, such as mathematics; the development of these brain areas can be identified through the shape of the skull (I).

69. Without a brain, consciousness is not possible (C).
70. The bigger the brain, the more intelligent the animal (I).

Neuromyths assertions are presented in *italic*.
C = correct
I = incorrect
